# Prevalence characteristics of high-risk human papillomaviruses in women living in Shanghai with cervical precancerous lesions and cancer

**DOI:** 10.18632/oncotarget.8262

**Published:** 2016-03-22

**Authors:** Ying Gu, Chenyun Ma, Jue Zou, Yi Zhu, Rong Yang, Yan Xu, Yu Zhang

**Affiliations:** ^1^ Department of Gynaecology and Obstetrics, The Seventh People's Hospital of Shanghai University of TCM, The New Pudong District, Shanghai 200137, China; ^2^ Clinical Laboratory, The Seventh People's Hospital of Shanghai University of TCM, The New Pudong District, Shanghai 200137, China; ^3^ Department of Pathology, The Seventh People's Hospital of Shanghai University of TCM, The New Pudong District, Shanghai 200137, China

**Keywords:** high-risk human papillomaviruses, cervicitis, cervical intraepithelial neoplasia grade 1 to 3, invasive squamous cell carcinoma

## Abstract

A complete understanding of the natural history of infection with high-risk human papillomaviruses (HPVs) in cervical cancer requires data from regional and ethnic studies. The prevalence of high-risk HPVs was evaluated retrospectively in 2040 patients with cervicitis, 239 with cervical intraepithelial neoplasia grade 1 (CIN1), 242 with CIN2/3, and 42 patients with invasive squamous cell carcinoma (SCC) based on data from patients who visited our hospital between May 2013 and May 2015. The rates of high-risk HPV infection in patients with cervicitis, CIN1, CIN2/3, and invasive SCC were 40.8%, 74.9%, 70.2%, and 83.3%, respectively. The three most dominant HPV genotypes were HPV16, 58, and 52. HPV16 and HPV58 positivity in cervicitis, CIN1, CIN2/3, and SCC patients were 20.9% and 16.4%, 19.0% and 20.1%, 44.1% and 23.5%, and 60.0% and 31.4%, respectively. Compared to cervicitis, the odds ratios (ORs) for CIN2/3 in HPV16- and HPV58-positive patients were 2.99 (95% confidence interval [CI]: 1.32–4.33) and 1.56 (1.11–3.21), respectively; for SCC, the corresponding values were 5.68 (2.31–7.893) and 2.33 (1.41–3.87). Further identifying of carcinogenic HPVs and a fully aware of regional differences in HPV genotype distribution are tasks of top priority for cervical cancer control and prevention.

## INTRODUCTION

In 2012, the American Cancer Society, American Society for Colposcopy and Cervical Pathology, and American Society for Clinical Pathology jointly published screening guidelines for the prevention and early detection of cervical precancerous lesions and cervical cancer [1]. The new screening recommendations addressed patient age-specific screening strategies, including the use of cytology and high-risk human papillomavirus (HPV) testing, the management of patients who were HPV-positive and the screening intervals for those who were HPV-negative, the age at which screening could be stopped, and screening strategies for women vaccinated against HPV16 and HPV18 infections [2]. Cytology and high-risk HPV testing have improved the detection of invasive cervical cancer at its early stages [1, 2]. Together with the treatment of preinvasive lesions, these measures have reduced the overall mortality from squamous cell cervical cancer (SCC) [1, 2]. In 2014, the United States Food and Drug Administration (FDA) approved the first HPV test for use in primary cervical cancer screening. Thus, today, HPV DNA testing alone can be used by healthcare professionals to determine whether women ≥25 years of age should undergo additional diagnostic testing for cervical cancer [3, 4]. However, the FDA authorization has caused intense disagreement and controversy. A supportive study performed in the USA, in which 42,209 nonpregnant women 21 years of age and older underwent routine cervical cancer screening, showed that primary screening for HPV in women ≥25 years of age is as effective as a hybrid screening strategy that uses cytology in women 25–29 years of age and as effective as co-testing in women ≥30 years of age [5]. Another follow-up of four European randomized controlled trials showed that HPV-based screening provided 60–70% greater protection against invasive cervical carcinomas compared with cytology [6]. Opponents of this approach claim that cytology testing has been carried out for >50 years and its validity is well established [1, 2] whereas long-term data showing that cytology testing can be replaced by HPV DNA testing is rare. A further objection is that studies supporting HPV DNA testing alone adopted cervical intraepithelial neoplasia (CIN) grade 3 (CIN3) instead of cervical cancer to evaluate the performance of HPV DNA testing in cervical cancer screening [7].

Thus, whether HPV DNA testing alone suffices in the early detection of cervical precancerous lesions and cancer is still uncertain. The question is relevant not only with respect to the adoption of a cervical cancer screening strategy but also points out gaps in the current understanding of the natural history of HPV infection and the role of HPV in oncogenesis. Current opinion is that persistent cervical infection with high-risk HPVs is necessary for the development of cervical cancer; indeed, some epidemiologic data have shown that nearly 100% of patients with cervical cancer test positive for HPV [8]. HPV16 and HPV18 are the two most carcinogenic HPV genotypes, accounting for 55–60% and 10–15% of cervical cancers, respectively [8–10]. However, amount of data showed that HPV DNA positive rates never reached 100% in patients with cervical cancer [8–11]. For example, a recent retrospective multicenter study showed that 9%, 23%, and 25% of the women tested were negative for HPV DNA <1 year, 1–3 years, and 3–5 years, respectively, before cervical carcinoma was diagnosed histologically [11]. Moreover, the HPV genotype distribution differs regionally worldwide. In a meta-analysis that summarized global data, Clifford et al. [12] concluded that HPV16, 31, and 51 were the three most dominant HPV genotypes. Thus, the relationship between HPV infection and cervical cancer, a seemingly conclusive topic, may in fact require a re-examination.

Shanghai is one of the largest cities in China, with a population of more than 25 million. However, the prevalence characteristics of high-risk HPV in its female residents with cervical precancerous lesions and cancer have rarely been evaluated. In this study, high-risk HPV infection rates and HPV genotype distribution were evaluated in 2563 patients living in Shanghai who had been diagnosed with cervicitis, CIN1, CIN2/3, or invasive SCC.

## MATERIALS AND METHODS

### Study population

This study was conducted in accordance with the World Medical Association Declaration of Helsinki. The Review Board of the Ethics Committee of Medical Research at Shanghai 7^th^ Hospital (Shanghai, China) approved the study protocols. Written informed consent was obtained from all patients according to the guidelines of the Chinese National Ethics Regulation Committee. All patients were informed of their rights to withdraw consent personally or via kin, caretaker, or guardian.

3011 outpatients and inpatients visiting the Department of Gynecology, Shanghai 7^th^ Hospital, between May 2013 and May 2015 for cervical diseases, were recruited consecutively. The criteria for exclusion in the study were as follows: patients with adenocarcinoma; patients are currently pregnant; patients with history of total uterine or cervical resection; patients with prior chemotherapy or radiation treatment for cervical neoplasia; patients with previous physical treatment of the cervix or hormone treatment for cervical disease, or patients with known human immunodeficiency virus infection. Thus, the study population consisted of 2563 (85.1%) female patients with average age around 40, of these participates, 2040 with cervicitis, 239 with CIN1, 242 with CIN2/3, and 42 with invasive SCC, respectively. The age, clinical gynecological, cytological, and histopathologic data as well as HPV testing results of the study participants were recorded.

### Cytological and histopathologic examinations

All participants were screened by clinical gynecological and cytological examinations. For each patient, cervical samples for cytological examination were taken, a sterile cytobrush was inserted into the cervical os and rotated through 360°, the acquired cells were then followed the instruction of the BD SurePath™ liquid-based Pap test (Becton, Dickinson and Company, Franklin Lakes, NJ). Patients, with epithelial cell abnormalities of atypical squamous cells (ASC) of undetermined significance (US) or higher cytological grade, would undergo colposcopy examination and biopsy. As Wentzensen N. et al. described [13], four lesion-directed biopsies for each patient were obtained from distinct locations of epithelium that turned white on application of 5% acetic acid in the cervical transformation zone using a sterile baby Tischler biopsy forceps. For patients with less than four lesions, substitute biopsies were added targeting normal-appearing cervical transformation zone epithelium. For patients without acetowhite lesion, biopsy was performed at the 3, 6, 9, and 12 o'clock positions of the cervix. All biopsies were ranked by order of severity at the time of colposcopy. The specimens were processed using standard cytological and histopathological methods and evaluated by at least two certificated pathologists. The final pathological result for each patient was reviewed by the chief pathologist, any uncertainties regarding cytological and histopathologic results were resolved by discussion. The pathologists were blinded to clinical data and group division. Terminology for reporting results of cervical cytology followed the 2001 Bethesda System: Cervicitis was defined as inflammation of the cervix; CIN1 was mild cervical intraepithelial neoplasia; CIN2 was moderate to marked cervical intraepithelial neoplasia; and CIN3 was severe cervical intraepithelial neoplasia and carcinoma *in situ* [14]. Squamous cell carcinoma (SCC) was diagnosed according to the Protocol for the Examination of Specimens From Patients With Carcinoma of the Uterine Cervix recommended by the College of American Pathologists [15].

### HPV genotyping

HPV DNA testing and genotyping are routinely performed in patients visiting the Department of Gynecology of our hospital. Before colposcopy, cervical specimens for HPV testing were also collected using a sterile cytobrush as the cell collecting procedures in cytological examination. The DNA in the specimen was extracted by using a QIAamp DNA Mini Kit (QIAGEN, Germany), HPV DNA testing and genotyping were done using a commercial detection kit provided by Hybribio (Chaozhou, China). The kit detects and distinguishes among 15 high-risk HPV strains (HPV16, 18, 31, 33, 35, 39, 45, 51, 52, 53, 56, 58, 59, 66, and 68) and 6 low-risk HPV strains (HPV6, 11, 42, 43, 44 and CP8304). HPV detection by the kit is achieved using polymerase chain reaction followed by HPV genotype-specific DNA microarray analyses. The performance of the HPV DNA testing and genotyping kit was approved by the China Food and Drug Administration. All detection procedures were carried out according to the protocols provided by manufacturers.

### Statistical analysis

Statistical analysis was performed using SPSS version 13.0 (SPSS, Chicago, IL). Continuous variables are presented as means ± standard deviations (SDs) and categorical data are presented as numbers (percentages). Differences between groups were examined using the *t* test, one-way ANOVA, χ^2^, or Fisher's exact probability test according to the characteristics of the data distribution. The odds ratio (OR) and relative 95% confidence interval (CI) were calculated using SPSS version 13.0. The significance level α was set at 0.05.

## RESULTS

### Characteristics of the patients

As shown in Table 1, the average ages of patients with cervicitis, CIN1, CIN2/3, and invasive SCC were 40.9 ± 12.3, 38.9 ± 11.6, 41.3 ± 10.9, and 44.5 ± 11.6 years, respectively; a trend towards an increase in the average age in patients with cervicitis to those with invasive SCC was noted, except for CIN1 patients, who were the youngest among the four groups. Patients with invasive SCC were significantly older than those in the other groups. The rates of high-risk HPV infection in patients with cervicitis, CIN1, CIN2/3, and invasive SCC were 40.8%, 74.9%, 70.2%, and 83.3%, respectively (Table 1). Although there were no statistically significant differences among the CIN1, CIN2/3, and invasive SCC groups, the infection rates in all three were significantly higher than that in the cervicitis group (Table 1). Because the data from the Centers for Disease Control and Prevention showed that the incidence of SCC increased sharply in women older than 30 years [16], all patients were divided into two groups (≤30 years and >30 years of age) to determine whether the distribution of HPV infection significantly differed between the two groups (Table 1), but this was not the case in our population.

**Table 1:** Clinical characteristics of the study patients (n = 2562)

Clinical parameter	Cervicitis (n = 2040)	CIN1 (n = 239)	CIN2/3 (n = 242)	Invasive SCC (n = 42)
Age	40.9 ± 12.3	38.9 ± 11.6	41.3 ± 10.9	44.5 ± 11.6*
High-risk HPV positivity in patients ≤ 30 years	183 (40.6%)	40 (70.2%)	22 (68.8%)	4 (100%)
High-risk HPV positivity in patients > 30 years	650 (40.9%)	139 (76.4%)	148 (70.5%)	31 (81.6%)
Overall HPV-positive rate	833 (40.8%)	179 (74.9%)**	170 (70.2%)**	35 (83.3%)**

CIN, cervical intraepithelial neoplasia; SCC, squamous cell carcinoma; HPV, human papillomavirus. Differences between groups were assessed using the χ2 test, Fisher's exact probability test, or a t-test according to the characteristics of the data distribution.

* P<0.01 vs. the cervicitis, CIN1, or CIN2/3 group;

** P<0.01 vs. the cervicitis group. Percentages for co-infections with two or more high-risk HPV strains were calculated separately for each one.

### Prevalence of high-risk HPVs

To determine the prevalence of high-risk HPV and of the different viral genotypes in our patients, they were screened for 15 high-risk HPVs. As shown in Table 2, all of the tested HPVs were highly prevalent in the study population except HPV81, which is rare. The three most prevalent HPV genotypes were HPV16, 58, and 52. Specifically, 20.9% of the patients with cervicitis, 19.0% with CIN1, 44.1% with CIN2/3, and 60.0% with invasive SCC were infected with HPV16. The infection rates for this genotype were significantly higher in CIN2/3 and invasive SCC patients than in cervicitis patients (Table 2). HPV58 infection was detected in 16.4%, 20.1%, 23.5% and 31.4% of the patients with cervicitis, CIN1, CIN2/3, and invasive SCC, respectively. The infection rates for this genotype were also significantly higher in CIN2/3 and invasive SCC patients than in cervicitis patients (Table 2). HPV52 infection was detected in 19.4%, 22.3%, 18.21%, and 11.4% of patients with cervicitis, CIN1, CIN2/3, and invasive SCC, respectively. None of the differences between groups were significant (Table 2). We also calculated the percentages of study patients co-infected with two or more high-risk HPV strains. Multiple infections are common [10] and were detected in 23.5% of our study population. However, there was no evidence in our patients that infection with multiple strains of HPV increased the risk of SCC.

**Table 2:** Prevalence of high-risk HPV in the study group

Genotype	Cervicitis (n = 833)	CIN1 (n = 179)	CIN2/3 (n = 170)	Invasive SCC (n = 35)
16	**174 (20.9%)**	**34 (19.0%)**	75 (44.1%)*	21 (60.0%)*
18	51 (6.1%)	13 (7.3%)	10 (5.9%)	0 (0%)
31	56 (6.7%)	17 (9.5%)	12 (7.1%)	2 (5.7%)
33	80 (9.6%)	23 (12.8%)	23 (13.5%)	1 (2.9%)
35	17 (2.0%)	5 (2.8%)	3 (1.8%)	0 (0%)
39	56 (6.7%)	17 (9.5%)	15 (8.8%)	1 (2.9%)
45	24 (2.9%)	5 (2.8%)	3 (1.8%)	1 (2.9%)
51	64 (7.7%)	20 (11.2%)	4 (2.4%)	1 (2.9%)
52	**162 (19.4%)**	**40 (22.3%)**	**31 (18.2%)**	**4 (11.4%)**
53	97 (11.6%)	9 (5.0%)	13 (7.6%)	0 (0%)
56	31 (3.7%)	3 (1.7%)	3 (1.8%)	0 (0%)
58	**137 (16.4%)**	**36 (20.1%)**	40 (23.5%)*	11 (31.4%)*
59	26 (3.1%)	5 (2.8%)	1 (0.6%)	0 (0%)
66	37 (4.4%)	11 (6.1%)	2 (1.2%)	1 (2.9%)
68	63 (7.6%)	13 (7.3%)	5 (2.9%)	0 (0%)
81	0 (0%)	0 (0%)	4 (2.4%)	0 (0%)

The top three dominant HPV genotypes were marked in bold. HPV, human papillomavirus; CIN, cervical intraepithelial neoplasia; SCC, squamous cell carcinoma.

* P<0.05 vs. the cervicitis group. Percentages for co-infections with two or more high-risk HPV strains were calculated separately for each one.

### ORs for HPV16 and 58 infections

Because HPV16 and HPV58 infections were significantly higher in patients with CIN2/3 and invasive SCC, we calculated the ORs and 95% CIs of such infections (Table 3). Compared to cervicitis, the ORs for CIN2/3 in HPV16- and HPV58-positive patients were 2.99 (1.32–4.33) and 1.56 (1.11–3.21), respectively. The corresponding values for invasive SCC in HPV16- and HPV58-positive patients were 5.68 (2.31–7.893) and 2.33 (1.41–3.87).

**Table 3:** ORs of HPVs for CIN2/3 and SCC

HPV16			HPV58		
OR	95% CI	P	OR	95% CI	P
*OR for CIN2/3*					
2.99	(1.32 ~ 4.33)	<0.001	1.56	(1.11 ~ 3.21)	0.026
*OR for Invasive SCC*					
5.68	(2.31 ~ 7.89)	<0.001	2.33	(1.41 ~ 3.87)	0.036

HPV, human papillomavirus; CIN, cervical intraepithelial neoplasia; SCC, squamous cell carcinoma; OR, odds ratio; CI, confidence interval.

### Prevalence of high-risk HPV in CIN1, CIN2/3, and SCC patients

To learn a more accurate estimate of HPV prevalence in CIN1, CIN2/3, and SCC patients world widely, we summarized the representative published data. As shown in Table 4, the ATHENA study and Wentzensen et al. showed that 62.0% and 68.9% of CIN1 patients were infected with high-risk HPVs [13, 17]; this prevalence is lower than that determined in our study. For CIN2/3 patients, the ATHENA study and Wheeler et al. [17, 18] showed that 82.0–97.1% were positive for high-risk HPV; this prevalence is higher than that calculated for our patients. For patients with SCC, the data on high-risk HPV positivity varies greatly across studies. In three recent Chinese reports [19–21], the rates of high-risk HPV infection were 91.1%, 85.3%, and 95.0%; the rate reported from a Shanghai study (85.3%) was close to that in our patients. According to the remaining researched data, which included single reports and two meta-analyses [9, 10, 12, 18, 22, 23], positivity ranged from 78.4% to 97.9% (Table 4). None of the data showed that 100% of patients with cervical cancer are HPV-positive despite the cross-sectional and prospective designs of the studies.

**Table 4:** Comparison of high-risk HPV positivity in this and other studies

Source	Region	Number of cases	HPV positive rate
*CIN1*			
Our data	Shanghai, China	239	74.9%
Castle PE. et al. [17]	USA	590	62.0%
Wentzensen N. et al. [13]	USA	170	68.9%
*CIN2/3*			
Our data	Shanghai, China	242	70.2%
Wheeler CM. et al. [18]	USA	1,213	97.1%
Castle PE. et al. [17]	USA	411	82.0 ~ 92.0%
*SCC*			
Our data	Shanghai, China	42	83.3%
Liang H. et al. [19]	Beijing, China	112	91.1%
Tao X. et al. [20]	Shanghai, China	449	85.3%
Zheng B. et al. [21]	Guangzhou, China	364	95.0%
Wheeler CM. et al. [18]	USA	808	91.0%
Bhatla N. et al. [22]	India	423	97.9%
Clifford GM. et al. [12]	Worldwide	87,337	89.5%
Munñoz N. et al. [10]	Spain	159	82.4%
Munñoz N. et al. [10]	Colombia	111	78.4%
Li N. et al. [23]*	Worldwide	26,667	90.9%
de Sanjose S. et al. [9]	Worldwide	9,486	87.0%

CIN, cervical intraepithelial neoplasia; SCC, squamous cell carcinoma; HPV, human papillomavirus.

* includes HPV genotype 16, 18, 58, 33, 45, 31, 52, 35, 59, 39, 51, 56, 68, 11, 53, 73, 6, 66, 70, 67, 82, 69, 26, 34, 30 and 85.

As the genotype distribution of high-risk HPVs differed regionally [24], we summarized the dominant HPV genotypes reported in representative published data. According to the integrated data from five studies, HPV16, 18, and 45 are the dominant genotypes in SCC patients [9, 10, 18]. In another two studies based on integrated data, HPV16, 18, and 33 and HPV 16, 18, and 58 were the three most dominant genotypes respectively [12, 23]. In our patients, HPV16, 58, and 52 were the high-risk HPV genotypes most commonly detected, which, differed from other reports [9, 10, 12, 18, 23], but consistent with the characteristics of local HPV infection [24].

## DISCUSSION

Our data showed that 40.8% of patients with cervicitis, 74.9% with CIN1, 70.2% with CIN2/3, and 83.3% with invasive SCC were positive for high-risk HPV DNA. HPV infection rates were significantly higher in CIN1, CIN2/3, and invasive SCC patients than in cervicitis patients, but they never reached 100%. Although single-center cross-sectional data do not reflect the real natural history of HPV infection in the population, our results suggest that HPV DNA testing alone might miss patients with cervical cancer or precancerous lesions. In addition, in our population, HPV genotypes 16, 58, and 52 were the dominant high-risk HPVs in patients with cervicitis, CIN1, CIN2/3, and invasive SCC, unlike in other reports [9, 10, 12, 18] in which HPV16, 18, and 45, HPV16, 18, and 33 or 16, 18, and 58 were most commonly detected. Despite the small sample size of our SCC group, the dominant genotypes remained stable across the cervicitis, CIN1, and CIN2/3 groups, which supports the credibility of our data. HPV16 and 58 were significantly associated with CIN2/3 and SCC, with ORs of 2.99 and 5.68 and 1.56 and 2.33 respectively. The absence of a similar significant association between HPV18 and either cervical cancer or precancerous lesions may have been due to small the size of the SCC group in our study, no adenocarcinoma patients or to our use of cervicitis patients, rather than healthy controls, as the basis of comparison in the calculations of the ORs.

In our group of SCC patients, the 83.3% positivity for HPV DNA was lower than the rates reported in most other studies [9, 10, 12, 18, 19, 21-23]. While, again, this low rate might have been due to the small size of the SCC group, our data are consistent with those in another report from Shanghai of 449 patients with SCC, in which the rate of HPV DNA positivity was 85.3%. Interestingly, although the overall positivity is lower in SCC patients, the positivity of HPV16 infection in SCC patients is 60.0% which consistent with most reports [7–10]. These evidences suggested that the performance of HPV detection kit is credible and our data likely reflect the real association pattern between HPV infection and cervical cancer or precancerous lesions in local area.

Because our study was cross-sectional, it was not possible to investigate the pathophysiological process of HPV infection and the development of cervical cancer or precancerous lesions in our patients with cervicitis, CIN1, CIN2/3, and invasive SCC. Therefore, we did not calculate the morbidity associated with the latter three conditions. Nonetheless, the diagnosis of cervical cancer or precancerous lesions was based on standardized histopathology, not cytopathology, which ensured the validity of our data. In this report, we only focused on patients with SCC but excluded patients with adenocarcinoma because the later is very rare in our clinical practice. So we could not know the prevalence characteristics of high-risk HPV in women living in Shanghai with cervical adenocarcinoma, because reports showed that cervical adenocarcinoma displayed specific HPV infection pattern [20,23]. In our previous report, 20.6%, 14.9%, 15.9%, 17.4%, and 21.2% of subjects in the age ≤ 24, 25–34, 35–44, 45–55, and > 55 groups, were high-risk HPVs positive, respectively [24]. High-risk HPVs infection displayed a inexplicable age difference, the data from the Centers for Disease Control and Prevention showed that the incidence of SCC increased sharply in women older than 30 years [16], in this report, high-risk HPV infection rates in subjects ≤30 years and >30 years of age showed no significantly difference.

The distribution of dominant HPV genotypes showed obvious regional differences [24]. HPV18 is reported to be one of the two most carcinogenic HPV genotypes (HPV16 and 18), accounting for 10–15% of cervical cancers [8–10]. In our population, the prevalence of HPV18 in patients with cervicitis, CIN1, CIN2/3 and invasive SCC were 6.1%, 7.3%, 5.9% and 0%, respectively, the overall prevalence rate of HPV18 is very lower which consistent with a recent Korea report, in that report, only 6 (2.5%) out of the 243 high-risk HPV positive subjects showed HPV18 infection [25]. HPV52 is also considered as a high-risk genotype and is especially frequent in Northern America, Africa, and Asia [26, 27]. In our population, the prevalence of HPV52 in our patients with cervicitis, CIN1, CIN2/3 and invasive SCC were 19.4%, 22.3%, 18.2% and 11.4%, respectively. The prevalence of HPV52 in our population is largely higher than other reports, in which HPV52 prevalent rates ranged up to 2.4% in women with normal cytological findings [27] and were 3.6% in women with SCC [23]. It is worth noting that although the overall prevalence of HPV52 was high in our population, no statistical evidence showed significant difference of the distribution among patients with cervicitis, CIN1, CIN2/3 and invasive SCC. Our results provide important information regarding the control and prevention of HPV infection within a single geographic area. Currently available quadrivalent and bivalent vaccines might not have a enough protective effect against HPV infection because they are derived from virus-like particles prepared from the L1 proteins of HPV 6, 11, 16, and 18 [28–30], whereas, as demonstrated in our study population, genotypes 16, 58, and 52 are among the predominant HPVs. However, in 2014, a nonavalent vaccine containing HPV 6, 11, 16, 18, 31, 33, 45, 52, and 58 antigens was licensed by the FDA [29]. This vaccine covers the HPV genotypes that are also prevalent in Shanghai and its multi-genotype specificity should provide a broader protective effect.

Current HPV DNA tests are mainly focused on high-risk HPV16, 18, 31, 33, 35, 39, 45, 51, 52, 56, 58, 59, and 68 [6, 13, 23, 25, 26], in our study, we detected 15 high-risk HPV strains (HPV16, 18, 31, 33, 35, 39, 45, 51, 52, 53, 56, 58, 59, 66, and 68) and 6 low-risk HPV strains (HPV6, 11, 42, 43, 44 and CP8304). We are not sure whether other more HPVs were associated with cervical cancer. Our data raised two issues need to be mindful in HPV control and prevention: first, regional differences in HPV genotype distribution; second, the real carcinogenic HPV revealing.
